# Injury of Head, &c., Operation of Trephining

**Published:** 1845-12-01

**Authors:** 


*Injury of the Head without fracture of Skull—compression of the brain from effusion of blood—Trephining—Recovery.*—Under the foregoing caption, we find in the Illinois Journal for Oct. 1845, a report of a very interesting case by our friend and former colleague, *D. Brainard, M. D.*, Prof. of Surgery in the Rush Med. College. Our limits only enable us to give a condensed abstract. The patient after an injury by blows with a stick upon the head, gradually became comatose, for which, bleeding, and other measures were pursued without relief. Dr. B. being called at this juncture, found, on shaving the head, that no marks of the injury existed upon it, except one, very slight, just at the superior angle of the os occipitis. He decided to apply the trephine, and in determining the most suitable location he was guided by the two following circumstances: 1st, it was ascertained that entire loss of motion, and partial loss of sensation existed on the *right* side of the body: 2d, we quote his language, "repeated observation has shown us, that when the members of one side are paralyzed from an injury, it is when this has been inflicted upon the central, or superior parts of the opposite hemisphere of the cerebrum. It is known, also, that extensive effusions of blood from such causes most frequently take place from the middle, meningeal artery, or some of its principal branches." "Guided by these facts, we determined on trephining an inch above the left ear, and a little anterior to it."

On removing a circle of bone, a coagulum instantly protruded. "Pieces of this were removed with the finger, until several ounces had come away." Consciousness, and power of motion over the right side returned directly after the operation; his recovery was rapid, without any unfavorable symptoms.

The case is certainly highly creditable to the ability and surgical tact of Dr. Brainard. The principles which determined the selection of the spot upon which to apply the trephine, should be recollected.

